# A novel missense COL9A3 variant in a pedigree with multiple lumbar disc herniation

**DOI:** 10.1186/s13018-023-04481-2

**Published:** 2024-01-03

**Authors:** Lejian Jiang, Chenhuan Wang, Zhaoming Ye, Qingfeng Hu

**Affiliations:** 1 Department of Orthopedics, The Fourth Affiliated Hospital, Zhejiang University School of Medicine, N1# Shangcheng Road, Yiwu, 322000 Zhejiang Province China; 2 Department of Orthopedic Surgery, The Second Affiliated Hospital, Zhejiang University School of Medicine, Hangzhou, China; 3 Key Laboratory of Motor System Disease Research and Precision Therapy of Zhejiang Province, Hangzhou City, Zhejiang Province China; 4 Orthopedics Research Institute of Zhejiang University, Hangzhou City, Zhejiang Province China

## Abstract

*Trp3* allele in *COL9A3* gene has been widely studied in populations with intervertebral disc disease. We identified a novel pathogenic variant in *COL9A3* gene in a pedigree with multiple lumbar disc herniation (LDH). The proband was a 14-year-old boy who developed LDH at the L4/5 and L5/S1 spinal segments. His father, paternal aunt and grandfather were diagnosed with LDH at an age of 35, 30 and 23, respectively. By applying whole exome sequencing, a heterozygous missense variant (c.1150C > T, p.Arg384Trp) in *COL9A3* was identified. According to the ACMG guidelines, this variant is predicted to be pathogenic. In addition, prediction tools found COL9A3 protein of this variant a reduced stability, some changed charge properties, and an altered spatial conformation. Findings expanded the mutational spectrum of LDH and contributed to the understanding of *COL9A3* in the pathogenesis of LDH.

## Introduction

Lumbar disc herniation (LDH) is a common lumbar spinal disorder that leads to back and leg pain [1]. Reportedly, the incidence rate of LDH is 1–3% in general populations, adding heavy socio-economical burdens in communities [2]. Etiological studies revealed that LDH is a genetically predominant disorder [3]. A number of genes related to disc structural components, inflammatory factors, and matrix remodelling proteases have been found to be associated with the occurrence of LDH [4, 5]. For example, *COL9A3* gene (Collagen type IX α3) has been repeatedly identified in different populations [6].

Collagen IX is a heterotrimeric protein encoded by *COL9A1*, *COL9A2*, and *COL9A3* genes [7]. It serves as an organizing bridge, crosslinking collagens and non-collagenous components in articular cartilage and disc extracellular matrix (ECM) [8]. *COL9A3* gene locates in chromosome 20q13.3 and encodes the α3 (IX) chain of collagen IX, which is a component of nucleus pulposus (NP) [9]. An early study of *COL9A3* on disc diseases found that patients with the *Trp3* (tryptophan) allele, also known as c.307C > T, p.Arg103Trp (rs61734651), had 2.7-fold higher risks of being diagnosed with lumbar spinal disease [10]. The minor allele frequency (MAF) of this single nucleotide polymorphism (SNP) was less than 0.05 in multiple datasets, GnomAD_exome (0.047), ExAC (0.048) and 1000 Genomes (0.022). Later on, while some independent studies demonstrated that *Trp3* was statistically associated with the increased risk of lumbar disc diseases [11, 12], some others failed to relate it with disc diseases. For instance, the *Trp3* allele in *COL9A3* was found not clustered in lumbar disc degeneration [13], and the genotype of *Trp3* allele was not related to clinical symptoms [14]. In addition, a number of meta-analysis studies did not observe the association between the *COL9A3 Trp3* polymorphism and disc degeneration [15].

Since previous population-based case–control studies on *COL9A3* gene have obtained inconsistent results [11–15], the underlying pathogenic mechanism of *COL9A3* is not fully understood to date. Here, we reported a clinical pedigree of LDH with significant genetic characteristics and involvement of multiple lumbar discs. By applying whole exome sequencing (WES), we identified a novel pathogenic single nucleotide variant (SNV) of *COL9A3.* Our findings provide insights of understanding the function of *COL9A3* in the pathogenesis of LDH.

## Materials and methods

### Case reports

The proband in this study was a 14-year-old boy who had low back pain and numbness in the lower extremities for half a year. The father, who accompanied for his son’s consultation, had radiculopathy in the left lower extremity for over two decades. The proband’s paternal aunt and grandfather were also clinically diagnosed with symptomatic LDH, with available MR images for confirmation. No other skeletal abnormality was observed in this family on radiological images. Written consent for each family member was obtained. This study was approved by the authors’ institutional ethic board.

### Whole exome sequencing, variant identification and validation

After blood sample collection, genomic DNA of the proband, his father, paternal aunt and grandfather were extracted using the Blood Genome Column Medium Extraction Kit (Kangweishiji, China). The extracted DNA samples were subjected to quality controlling using Qubit 2.0 fluorimeter and electrophoresis with 0.8% agarose gel. Whole exome library was constructed using Roche Nimble Gen Seq EZ Exome Enrichment Kit V2.0 and Seq EZ Exome Enrichment Kit V2.0 capture probes (Roche, USA). High-throughput sequencing was performed on a Novaseq 6000 instrument (CHIGENE, Beijing, China) [16]. Quality control of whole exome sequencing data, variants calling and variant annotation was performed in the same institution.

Variant prioritization was performed based on guidelines released by ACMG (The American College of Medical Genetics and Genomics). First, co-segregation analysis was performed to exclude SNVs contradictory to the phenotypic data. SNVs were analysed under the assumptions of Mendelian dominant inheritance, recessive inheritance and sex-linked inheritance. Second, only rare variants with MAF < 1% were included for further selection [17, 18]. Candidate variants were selected based on the 1000 Genomes (https://www.internationalgenome.org), the Exome Sequencing Project (ESP), the Exome Aggregation Consortium (ExAC, http://exac.broadinstitute.org), Allele Frequency Aggregator (ALFA) and the Genome Aggregation Database (gnomAD, https://gnomad.broadinstitute.org). Third, synonymous variants and non-coding region variants were excluded. In silico prediction tools were applied to analyse pathogenicity of identified missense variants (*Sorting Intolerant From Tolerant (SIFT)*, *Polymorphism Phenotyping v2 (Polyphen2)*, *Multivariate Analysis of Protein Polymorphism (MAPP)*, *Mutation Taster*, *Mendelian Clinically Applicable Pathogenicity (M-CAP)*, *Rare Exome Variant Ensemble Learner (REVEL)* and *Combined Annotation Dependent Depletion (CADD)*) [19–25]. The deleterious effects of splice variants were predicted by *MaxEntScan* and *dbscSNV* [26, 27]. Next, evolutionary conservatism was analysed by *phastCONS*, *phyloP* and *Genetic Evolutionary Rate Profiling (GERP)* [28–30]. At last, protein function, GO (Gene ontology) annotations, tissue-specific distribution and existing literature were searched to evaluate the remaining SNVs.

Sanger sequencing was performed to validate the identified candidate variants. Sanger sequencing was performed with these primers:

Forward primer 5′-CAGGCGTCCCTGTGAGTATC-3′,

Reverse primer 5′-CATCAAGGCAACCAAATGCCA-3′.

The RefSeq accession numbers of the transcript and the corresponding protein isoform of *COL9A3* we used for mutation nomenclature were NM_001853.4 and NP_001844.3, respectively.

## Results

### Clinical characterization of the pedigree with multiple lumbar disc herniation

**Fig. 1:**
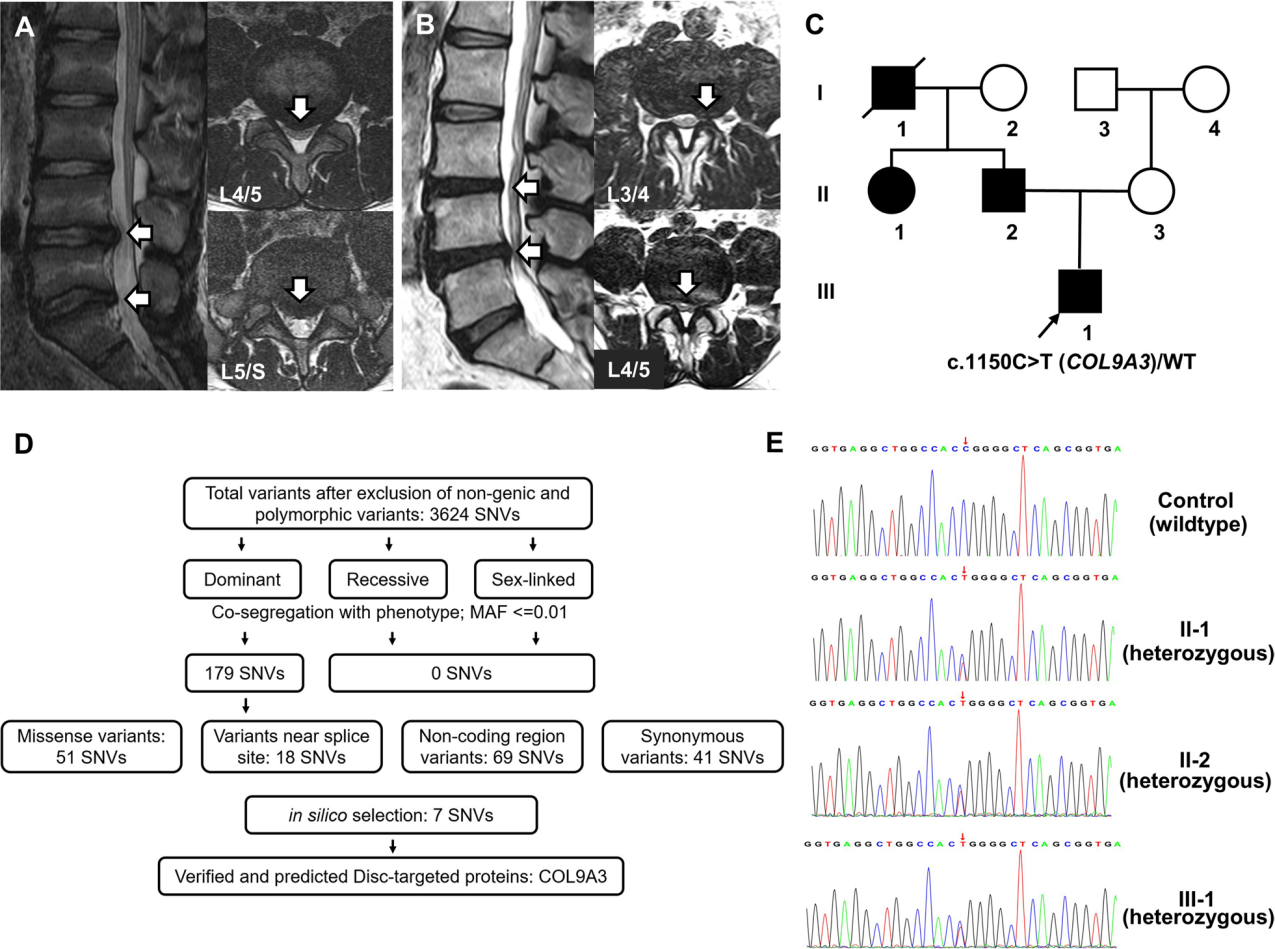
Magnetic resonance (MR) characteristics, pedigree chart and sequencing results of the proband and family members. **A** MR imaging of the proband: disc herniation at L4/5 and L5/S1 spinal segments. **B** MR imaging of the proband’s father: disc herniation at L3/4 and L4/5 spinal segments. **C** Pedigree chart: the proband’s paternal aunt and grandfather were diagnosed with lumbar disc herniation at an age of 30 and 23, respectively. **D** Summary of exome sequencing of the pedigree: the identified SNV (c.1150C > T, p.Arg384Trp) is located at *COL9A3*, a gene encoding one of the three alpha chains of type IX collagen. **E** Sanger sequencing: the heterozygous *COL9A3* missense variant (c.1150C > T, p.Arg384Trp) was confirmed

The proband (height 1.65 m, weight 55.6 kg) is a 14-year-old Chinese boy suffering from low back pain and numbness in the lower limbs for 6 months before consultation. Magnetic resonance (MR) imaging revealed LDH at L4/5 and L5/S1 spinal segments (Fig. 1A). Growth and development of the proband were normal in adolescence. The proband did not experience waist trauma or excessive physical labour within 6 months before the onset of clinical symptoms. Blood biochemistry measurements and radiographs of limbs did not present any abnormality on the proband. A follow-up investigation of the LDH-related clinical symptoms and radiographs was carried out on the proband’s immediate family members. The proband’s father, who suffered from left lower limb radiculopathy for decades, had L3/4 and L4/5 LDH based on his lumbar spine MR images (Fig. 1B). The proband’s father, paternal aunt and grandfather had back pain and radicular leg pain, which were consistent with MR findings. They were clinically diagnosed with LDH at an age of 35, 30 and 23, respectively (Fig. 1C). None of them reported a history of waist injury before the onset of LDH‐related symptoms nor any other abnormality in the musculoskeletal system, except for the proband’s grandfather, who had degenerative kyphosis after 60 years old.

### Identification of a novel *COL9A3* missense variant in the LDH family

We performed WES from four members (proband III-I, affected father II-2 and paternal aunt II-1, and unaffected mother II-3) to identify the candidate gene for the phenotypic manifestation of LDH. The work flow is summarized in Fig. 1D. A total of 3624 SNVs were identified after exclusion of non-genic and polymorphic variants. SNVs which did not co-segregate with the phenotype and were more than 1% frequency in the public genomes databases were excluded. Under the assumption of Mendelian dominant inheritance, 179 SNVs were identified. Synonymous variants and variants in non-coding region were then excluded, resulting in 69 SNVs. Besides, several in silico prediction tools invariably predicted that seven of the SNVs might cause damage to the protein, and no SNV near splice site had deleterious effects on the protein. In view of the protein function, GO annotations, tissue-specific distribution and the facts in published literatures, a novel heterozygous missense variant (c.1150C > T, p.Arg384Trp) in *COL9A3* was identified, and further confirmed by Sanger sequencing (Fig. 1E).

The MAF of this SNP was less than 0.01 in different databases, ESP (0.00), 1000 Genomes (0.00), ALFA (0.00), GnomAD_exome (0.00004) and ExAC (0.00003). This variant was predicted to be pathogenic by in silico prediction tools, Provean (3.16), SIFT (0.005), Polyphen2_HDIV (1.0), MutationTaster (0.999494), M-CAP (0.705) and REVEL (0.725). In addition, with a CADD score > 20, this variant was also evaluated to be deleterious in GERP, phyloP and phastCons software. According to the variant interpretation guideline of ACMG (PM2, PP1, PP2, PP3 and PP4), this variant (c.1150C > T) was classified as “likely pathogenic” variant [31].

### Change of charge properties and instability of COL9A3 protein with p.Arg384Trp variant

**Fig. 2:**
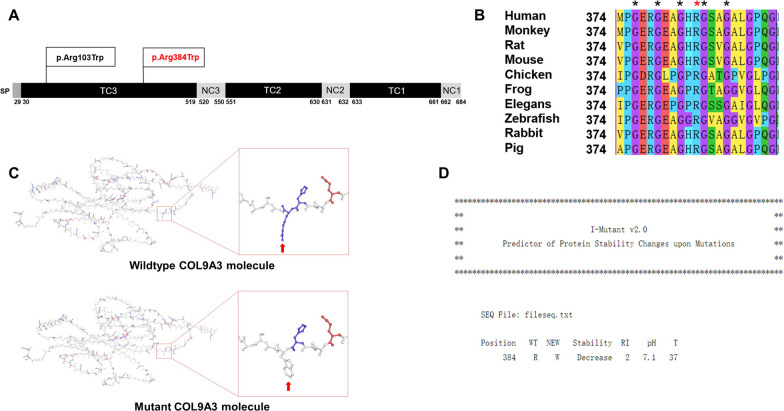
An illustration of COL9A3 protein structure, Pathogenic variants, Sequence alignment among multiple species and Prediction results of *COL9A3* missense variant (c.1150C > T, p.Arg384Trp). **A** The identified *COL9A3* variant (c.1150C > T, p.Arg384Trp, Red) locates within the third collagenous region, resulting in the substitution of arginine (Arg) with tryptophan (Trp) in the amino acid sequence. Previously identified variant (p.Arg103Trp) were marked in black. **B** Multiple species sequence alignment using MEGA11 software shows that this variant is highly conserved among common species. **C** Protein structure predicted by Swiss-Model reveals an altered protein structure and a charge property change by the replacement of 384th Arg residue. The 384th amino acid is mutated from a basic amino acid (Arg, Blue) to an aromatic hydrophobic amino acid (Trp, White). **D** The I-Mutant v2.0 software reveals that the variant can result in decreased COL9A3 protein stability at optimal pH and temperature. SP: Signal peptide; TC: Triple-helical collagenous region; NC: Non-helical collagenous region; WT: Amino acid in Wild-Type Protein; NEW: New Amino acid with the variant; RI: Reliability Index; pH: − log [H+]; T: Temperature in Celsius degrees.

The p.Arg384Trp variant is situated within the collagenous domain, resulting in the substitution of arginine with tryptophan in amino acid sequence (Fig. 2A). Protein sequence alignment revealed that this locus is highly conserved among common species (Fig. 2B). Furthermore, the impacts of the p.Arg384Trp variant on the structure, function, and stability of COL9A3 were analysed using Swiss-Model (Fig. 2C) [32–34]. According to the prediction, the variant at this site changed the charge property and hydrophilicity of COL9A3 protein due to the substitution of arginine (Basic amino acid) with tryptophan (Aromatic hydrophobic amino acid). In addition, the prediction result in I-Mutant2.0 revealed a decreased stability of COL9A3 protein (Fig. 2D) [35].

## Discussion

Pedigree-based screening can identify pathogenic genes for heritable LDH. In this study, LDH patients were characterized by early-aged onset, multi-segment disc involvement, dominant inheritance, and the absence of extra-discal deformities in the musculoskeletal system. The clinical significance of the identified *COL9A3* variant (c.1150C > T, p.Arg384Trp) has not been previously reported. Our findings suggested that this variant is disease-causing rather than a susceptibility factor for LDH.

Collagen IX plays an important role in forming and stabilizing the collagen matrix in the disc. SNPs that result in tryptophan polymorphisms in collagen IX genes, such as *Trp2* allele (p.Gln326Trp) in *COL9A2* and *Trp3* allele in *COL9A3*, have been linked to an increased risk of lumbar disc disease in different populations [10, 36]. We summarized the phenotypes, sampling regions, sample sizes and statistical results of *COL9A3* pathogenic variants in intervertebral disc diseases (Table 1) and found that results from different studies were inconsistent. In previous research, *Trp3* variant (c.307C > T, p.Arg103Trp) was the only pathogenic variant site identified in *COL9A3* among populations (Fig. 2A).

**Table 1:** Summary of identified COL9A3 variants and related information in disc diseases

Mutation	Phenotype	Region/race	Sample size	Significance	Conclusion	References
p.Arg103Trp	Disc disease*	Finnish	492	Yes	Trp3 allele frequency was 12.2% in patients	Paassilta et al. [10]
p.Arg103Trp	Disc degeneration	Finnish	135	Yes	Trp3 allele frequency was 17% in patients	Solovieva et al. [37]
p.Arg103Trp	Disc disease	American	14	No	Trp3 allelic protein has no obvious effect on disc disease	Matsui et al. [38]
p.Arg103Trp	Disc degeneration	Finnish	85	No	Trp3 allele alone is not likely to cause disc degeneration, but it may be one of the predisposing factors	Noponen et al. [39]
p.Arg103Trp	Disc disease	Greek	105	No	Trp3 allele in *COL9A3* is likely to be less significant susceptibility factors for intervertebral disc disease	Kales et al. [40]
p.Arg103Trp	Disc degeneration	Chinese	804	No	The Trp3 allele was absent from the Southern Chinese population	Jim et al. [36]
p.Arg103Trp	Disc degeneration	Finnish	135	No	The effect of the *COL9A3* polymorphism on disc degeneration maybe modified by *IL-1β* polymorphism	Solovieva et al. [41]
p.Arg103Trp	Disc degeneration	Japanese	84	No	No patients had the Trp3 allele	Higashino et al. [42]
p.Arg103Trp	Disc disease	Finnish	211	No	Trp3 allele had less association with disc disease phenotype	Virtanen et al. [43]
p.Arg103Trp	Disc disease	Finnish	228	No	Trp3 allele had less association with disc disease phenotype	Karppinen et al. [44]
p.Arg103Trp	Disc degeneration	American	133	Yes	The product of the Trp3 allele may cause degeneration of intervertebral discs	Zhu et al. [11]
p.Arg103Trp	Disc disease	Singaporean	54	No	The Trp3 allele was absent from all the subjects	Lim et al. [45]
p.Arg103Trp	Disc disease	Indian	100	No	Allelic variation in *COL9A3* was found to have no significant correlation with disc disease	Rathod et al. [46]
p.Arg103Trp	Disc degeneration	Southern European	100	Yes	Trp3 allele was associated with more severe disc degeneration based on Pfirrmann scores	Toktas et al. [47]
p.Arg103Trp	Disc degeneration	Iranian	165	Yes	Male patients with Trp3 allele were more likely to develop disc degeneration	Bagheri et al. [48]
p.Arg103Trp	Disc herniation	American	15	No	Collagen-encoding variants may be a genetic risk factor for lumbar disc herniation	Theodore et al. [49]
p.Arg103Trp	Disc herniation	Chinese	768	Yes	Trp3 allele significantly influence the risk of lumbar disc herniation	Yang et al. [50]
p.Arg384Trp	Disc herniation	Chinese	3	–	A novel heterozygous missense variant co-segregating with phenotypes, was predicted to be pathogenic	Current case

*Disc Disease: disc degeneration and herniation

To date, various mechanisms have been proposed to explain COL9A3 dysfunction leading to disc disease. For example, *Trp3* allele in *COL9A3* increases the proportion of tryptophan in the collagen, which subsequently alters the triple helical structure of the protein. This substitution may also disrupt the process of lysyl oxidase-catalysed crosslinking, increase the risk of disc instability, and eventually lead to the occurrence of disc diseases [37]. Also, silencing the expression of *COL9A3* can activate the MAPK pathway and downstream apoptosis-related factors, resulting in attenuated NP cells proliferation and promoted cell apoptosis [51]. In animal models, *Col9a3* deficient mice exhibit abnormalities in the disc and cartilage, including shortened body height, impaired maturation of articular cartilage, and calcified epiphyseal cartilage [52]. In addition, absence of collagen IX may be related to premature disc degeneration with annular lesions through disrupting the Ihh-PTHrP pathway in ageing mice [53].

Although *COL9A3* gene has been repeatedly studied, the underlying mechanism and its effects on the pathogenesis of LDH remain unclear. Results of this study further evidenced that *COL9A3* plays an important role in LDH. A new genetic variant was identified in this report, but further studies are needed to explore the pathogenesis of LDH with the *COL9A3* pathogenic variant (c.1150C > T, p.Arg384Trp) and develop possible treatment strategies.

## Conclusions

In this report, we identified a novel missense pathogenic variant, which is conserved among common species, in a family with multi-segment LDH. The identified *COL9A3* variant was predicted to have detrimental effects on the structure and stability of COL9A3. We provided new evidence to support an association between *COL9A3* pathogenic variants and LDH, and extended the mutational spectrum of LDH.

## Data Availability

Not applicable.
